# Performance comparison of large language models for medication counseling in people living with HIV

**DOI:** 10.3389/fpubh.2026.1850095

**Published:** 2026-05-29

**Authors:** Can Huang, Yanfang Sun, Meng Chen, Lin Zhang, Wei Liu

**Affiliations:** Beijing Youan Hospital, Capital Medical University, Beijing, China

**Keywords:** artificial intelligence, capability evaluation, HIV, large language models, medication consultation

## Abstract

**Objective:**

This study aimed to evaluate the comprehensive performance of five large language models (LLMs), namely ChatGPT, DeepSeek, Doubao, Kimi, and Qwen, in addressing medication consultation inquiries for people living with HIV (PLWH) in a Chinese-language context, thereby providing evidence for their clinical application and further model optimization.

**Methods:**

A total of 55 real-world medication consultation questions covering mainstream antiretroviral drugs for PLWH were screened and classified from Beijing Youan Hospital, Capital Medical University, a specialized infectious disease hospital in China. Five LLMs were queried within a fixed period, and expert evaluations were conducted across five dimensions: accuracy, relevance, completeness, clarity, and reliability.

**Results:**

The comprehensive scores ranked from highest to lowest were DeepSeek (4.47), Qwen (4.33), Kimi (4.24), Doubao (4.13), and ChatGPT (3.41), with highly significant differences were observed among all models (*H*=182.14, *p* < 0.001). Regarding dimensional scores, the ranking was clarity (4.53) > reliability (4.33) > completeness (4.13) > accuracy (3.95) > relevance (3.62). ChatGPT exhibited statistically significant differences compared with all other models (*p* < 0.001); no significant differences were found between DeepSeek and Qwen or between Doubao and Kimi (*p* > 0.05).

**Conclusion:**

Significant differences were observed in the capacity of the five LLMs to address medication consultations for PLWH within the Chinese-language context. DeepSeek and Qwen achieved optimal overall performance, Doubao excelled in clarity, whereas ChatGPT yielded the poorest results. All models demonstrated significant limitations when handling complex pharmaceutical inquiries and cannot fully replace professional clinical pharmacists. Further optimization focusing on high-quality medical domain dataset training and algorithm refinement is therefore warranted.

## Introduction

Acquired Immunodeficiency Syndrome (AIDS) is caused by infection with the Human Immunodeficiency Virus (HIV), and patients are required to receive standardized Antiretroviral Therapy (ART) for life (1). Medication adherence, avoidance of drug–drug interactions, and proper management of adverse reactions are the core links that determine treatment success or failure, reduce mortality, and lower the risk of complications (2–5). The current number of PLWH in China has exceeded 1.3 million (6), and patients continuously face high-frequency needs for medication consultation during long-term treatment, such as dosage and administration, missed dose remediation, safety of combined medications, and management of adverse reactions (7–9). Traditional models such as outpatient consultation, telephone follow-up, and pharmacist education have practical bottlenecks including insufficient resource supply, delayed response, and uneven coverage, making it difficult to meet patients’ individualized and round-the-clock medication guidance needs (10–12).

In recent years, large language models (LLMs) have been rapidly applied in medical scenarios such as chronic disease management, medication education, and prescription review, relying on their capabilities in natural language understanding, knowledge integration, and interactive response. They have become important auxiliary tools to alleviate the pressure of pharmaceutical services and improve patients’ self-management abilities (13–15). Existing studies have confirmed that LLMs can complete tasks such as medication consultation, health education, and risk prompting in fields such as diabetes, hypertension, breast cancer, and digestive system diseases, showing application potential in dimensions such as information completeness and expression clarity (16–19). However, HIV medication has specific characteristics such as complex regimens, widespread combined medication use, and rapid knowledge updates, which place higher requirements on the model’s medical accuracy, risk identification ability, and reliability of evidence-based basis (20, 21).

Currently, there are few comparative studies on the performance of LLMs in addressing medication consultations for PLWH (22, 23). Most evaluation dimensions mainly focus on the description of overall scores, lacking refined multi-dimensional decomposition and in-depth analysis of individual questions. Additionally, no studies have examined the performance of LLMs in scenarios such as drug–drug interactions, individualized dose adjustments, and medication use in special populations.

Based on this, this study relies on real HIV medication consultation cases from Beijing Youan Hospital, Capital Medical University (a specialized infectious disease hospital), selecting five mainstream LLMs in China (ChatGPT, DeepSeek, Doubao, Kimi, and Qwen). A standardized five-dimensional scoring system was adopted to systematically evaluate the comprehensive performance and dimensional differences of the models in HIV medication consultation. Combined with qualitative comparison of answers to 55 individual questions, the study analyzes the differences and characteristics of each model’s capabilities, providing a scientific basis for the precise application and algorithm optimization of LLMs in clinical pharmaceutical consultation.

## Materials and methods

### Consultation questions and classification

A total of 55 core medication consultation questions for HIV were selected from real medication consultation cases and medication education materials in Beijing Youan Hospital, Capital Medical University, a specialized infectious disease hospital, covering mainstream antiretroviral drugs such as Efavirenz (EFV), Raltegravir (RAL), and Bictegravir/Emtricitabine/Tenofovir Alafenamide (Biktarvy). These questions were classified into 5 primary categories and further subdivided into 8 secondary subcategories, including dosage and administration, adverse reactions, and drug–drug interactions. The distribution of the number of questions was as follows: 10 questions on medication administration methods, 25 questions on drug safety, 5 questions on basic drug information, 5 questions on medication use in special populations, and 10 questions on drug efficacy and medication adherence. The distribution of questions across these categories was intentionally designed to reflect the frequency of real-world medication consultations encountered in our clinical setting. Consequently, more common scenarios (e.g., general dosing) are represented by a larger number of questions, while more complex but less frequently encountered scenarios (e.g., special populations) have relatively fewer items. This approach ensures that the evaluation is grounded in the practical demands of clinical pharmacy practice (detailed in Supplementary Table S1).

### Selection of evaluation models

To test the current basic capability of LLMs in handling medication consultation and medication education in the Chinese context, this study selected several well-known and widely used models in China, and chose their free versions that are more accessible and usable for the general public for evaluation. The selected models included ChatGPT-5.2, DeepSeek-3.2, Doubao-2.0, Kimi-2.5, and Qwen-3.5. All models were accessed through their official public web interfaces rather than APIs. All queries and response collection were independently performed by two trained researchers. No external tools or plugins were used during the evaluation process, while internet access was enabled for all LLMs to support real-time information acquisition. All models generated responses using default parameter settings to ensure the consistency of test conditions. All queries were completed from March 2 to March 6, 2026, during which no public version updates of the used models occurred. Each question was queried only once, and the conversation was reset after each query to avoid memory bias. Finally, the answers to all questions were sorted out and summarized in a preset result table (detailed in Supplementary Table S2).

### Standardized prompt design

To ensure consistency and comparability across all models, a single standardized prompt was designed and applied identically to ChatGPT, DeepSeek, Doubao, Kimi, and Qwen throughout the experiment. No modifications or adjustments were made to prompts between models.

The standardized prompt used in this study was:

“Please answer the following HIV medication consultation question accurately, professionally, comprehensibly, and safely based on standard clinical guidelines and drug instructions: [Question text].”

This unified prompt ensured all models received identical task instructions and reduced potential bias caused by prompt variation.

### Scoring dimensions and criteria

Given the lack of a unified evaluation standard in the field, five core scoring dimensions were constructed with reference to international relevant indicators for information evaluation (24, 25), combined with the professional requirements of HIV medication consultation. A 5-point Likert scale (1–5 points) was adopted for evaluation. Two experts with more than 10 years of clinical pharmacy work experience served as raters, who independently scored the answers of the five models to 55 questions item by item and dimension by dimension without knowing the names of the models (blinded evaluation). For items with inter-rater disagreement of ≥1 point, a third reviewer (a chief pharmacist with 20 years of clinical pharmacy experience) was invited to make a ruling. Consensus was achieved for all items after adjudication, ensuring the objectivity and reliability of the scoring results (detailed in Supplementary Tables S3, S4).

The scoring dimensions included:

- Accuracy: the degree of consistency between the answer and clinical guidelines or drug instructions.
- Relevance: the degree of matching between the answer and the question without deviation.
- Completeness: the degree to which core information is not missing and key points are fully covered.
- Clarity: the degree to which the expression is organized, easy to understand, and actionable.
- Reliability: the degree to which the answer is based on authoritative evidence, logically rigorous, and free from the risk of errors.

### Inter-rater reliability analysis

To ensure the reliability of the expert evaluation process, all responses were independently scored by two senior clinical pharmacists. Inter-rater agreement was quantified using Cohen’s kappa coefficient, percentage agreement, and 95% confidence intervals (CI). Kappa values were interpreted as follows: <0.20 = slight, 0.21–0.40 = fair, 0.41–0.60 = moderate, 0.61–0.80 = substantial, and 0.81–1.00 = almost perfect agreement.

### Statistical methods

Data were entered using Microsoft Excel 2016 and analyzed with R software (Version 4.5.2). Continuous variables were described as mean ± standard deviation (*x̄* ± s) and median (M). After testing for normality and homogeneity of variance, non-parametric data were analyzed using the following statistical methods:

- Overall comparison between groups: Kruskal–Wallis *H* test.
- Post-hoc multiple comparisons: Dunn’s test with Bonferroni correction; − Comparison between dimensions: Friedman test.
- Correlation analysis: Spearman’s rank correlation test.
- A two-tailed *p*-value < 0.05 was considered statistically significant.

### Qualitative content analysis

To establish a robust gold standard for evaluation, reference answers for all 55 questions were collaboratively developed by two chief clinical pharmacists with over 20 years of experience in HIV care. These reference answers were meticulously constructed based on the most current clinical evidence, primarily drawing from the Chinese Guidelines for the Diagnosis and Treatment of AIDS (2024 Edition) and the official package inserts of the medications as provided by the hospital pharmacy. The development process involved a rigorous review to ensure accuracy, completeness, and alignment with real-world clinical practice. Any discrepancies were resolved through discussion and consensus, ensuring the reliability of the reference answers used for subsequent model evaluation. Using these reference answers as the gold standard, a question-by-question qualitative comparative analysis of the answers from the five models was conducted (26, 27). The core characteristics and differences of each model in terms of answer content, expression style, logical reasoning, and clinical operability were summarized, with a focus on analyzing the following aspects: matching degree of core points, types of errors, ability to decompose complex problems, clinical operability, and comprehensibility for patients.

## Results

### Inter-rater reliability

Inter-rater reliability was assessed across a total of 1,375 scoring entries (55 questions × 5 models × 5 dimensions). The overall exact agreement rate between the two raters was 86.62%, with 1,191 consistent scores and 184 inconsistent scores. Overall, unweighted kappa = 0.799 (95% CI: 0.775–0.825, *p* < 0.001), linear weighted kappa = 0.870 (95% CI: 0.852–0.888, *p* < 0.001), and quadratic weighted kappa = 0.931 (95% CI: 0.918–0.942, *p* < 0.001). All kappa values were statistically significant, indicating almost perfect overall agreement between the two evaluators. Notably, Accuracy showed the highest consistency (*κ* = 0.881), whereas Clarity exhibited slightly lower but still substantial agreement (*κ* = 0.644), consistent with the subjective nature of readability assessment (detailed in Supplementary Table S5).

### Comparison of comprehensive scores of LLMs

The comprehensive scores of the five LLMs ranked from highest to lowest were as follows: DeepSeek, Qwen, Kimi, Doubao, and ChatGPT (Table 1). There were extremely significant statistical differences in the comprehensive scores among the five models (*H* = 182.14, *p* < 0.001). Multiple comparison results showed that ChatGPT had statistically significant differences compared with the other four models (*p* < 0.001); there were no statistically significant differences between DeepSeek and Qwen, as well as between Doubao and Kimi (*p* > 0.05). Table 1 for details.

**Table 1:** Comprehensive scores and descriptive statistics of models.

Rank	Model	Mean value	Standard deviation	Median
1	DeepSeek	4.47	0.87	5.00
2	Qwen	4.33	1.03	5.00
3	Kimi	4.24	1.01	5.00
4	Doubao	4.13	1.07	4.00
5	ChatGPT	3.41	1.18	4.00

### Scores of each evaluation dimension

**Figure 1:**
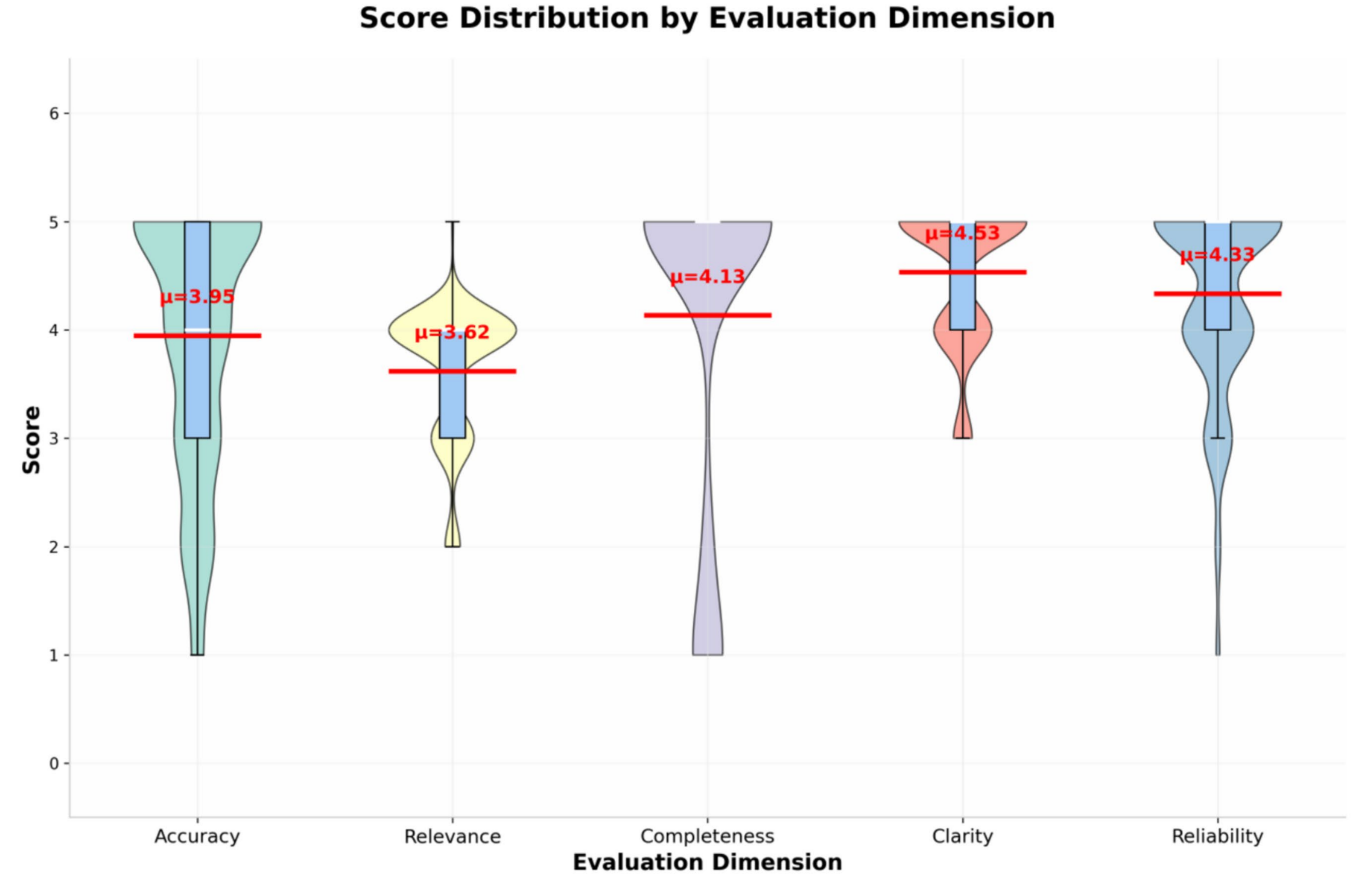
Violin plot of scores for the five evaluation dimensions. The figure shows the distribution pattern and dispersion degree of scores for each dimension.

There were statistically significant differences in the scores among the five evaluation dimensions (*χ*^2^ = 112.97, *p* < 0.001), and the mean score for each dimension was ranked as follows: clarity > reliability > completeness > accuracy > relevance (Table 2). Figure 1 shows that the completeness dimension had the largest distribution range with more low scores, serving as a key dimension to distinguish the capabilities of different models; the overall scores of the accuracy, clarity, and reliability dimensions were concentrated between 3 and 5 points, with a median of approximately 4 points, indicating good performance but existing individual differences. The relevance dimension had the most concentrated distribution but the lowest scores.

**Table 2:** Dimension-specific performance scores of five LLMs (values presented as mean ± standard deviation).

Evaluation dimension	ChatGPT	DeepSeek	Doubao	Kimi	Qwen	Dimension average
Clarity	3.91 ± 0.29	4.76 ± 0.60	4.87 ± 0.33	4.24 ± 0.85	4.89 ± 0.31	4.53 ± 0.66
Reliability	3.71 ± 1.09	4.62 ± 0.80	4.35 ± 0.64	4.44 ± 0.78	4.56 ± 0.68	4.33 ± 0.88
Completeness	3.38 ± 1.92	4.91 ± 0.35	3.91 ± 1.72	4.15 ± 1.55	4.33 ± 1.38	4.13 ± 1.57
Accuracy	2.78 ± 0.99	4.31 ± 1.14	3.93 ± 0.97	4.44 ± 0.95	4.29 ± 1.12	3.95 ± 1.20
Relevance	3.25 ± 0.44	3.73 ± 0.67	3.60 ± 0.56	3.93 ± 0.46	3.58 ± 0.80	3.62 ± 0.64

### Model-dimension cross performance

**Figure 2:**
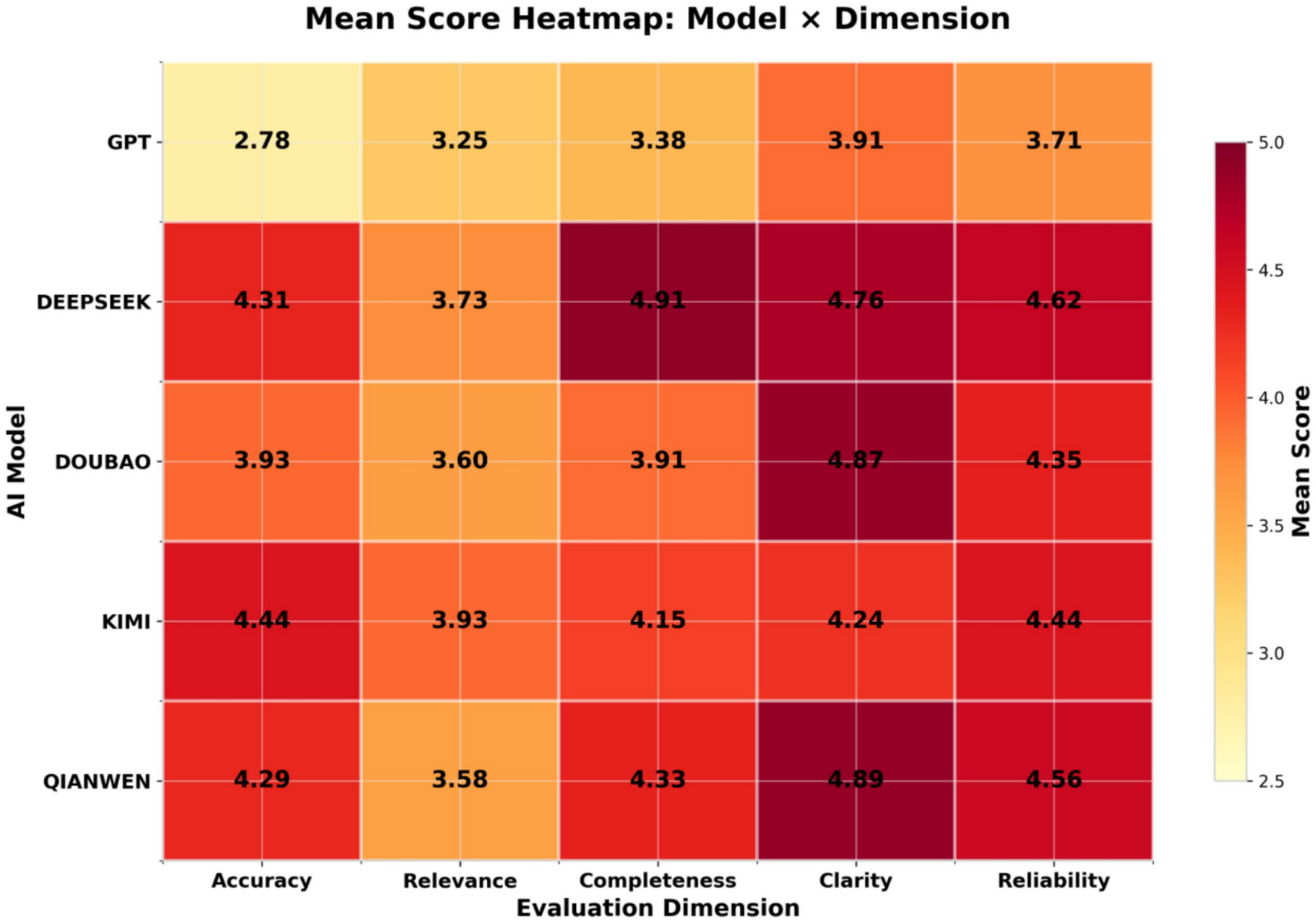
Heatmap of model-dimension cross scores: the darker the color, the higher the score, which clearly shows the advantages and shortcomings of each model in different dimensions.

The heatmap (Figure 2) shows that each model exhibited differentiated advantages in different dimensions: DeepSeek led in the dimensions of completeness (4.91) and reliability (4.62); Qwen performed excellently in the dimensions of clarity (4.89) and reliability (4.56), ranking in the first echelon together with DeepSeek; Kimi achieved the highest score in the accuracy dimension (4.44); Doubao performed well in the clarity dimension (4.87) but was relatively weak in the relevance dimension (3.60); ChatGPT had the lowest scores in all dimensions, with a particularly prominent shortcoming in accuracy (2.78). Overall, all models performed generally best in the clarity dimension and relatively poorly in the relevance dimension.

### Score differences among different topic categories

**Figure 3:**
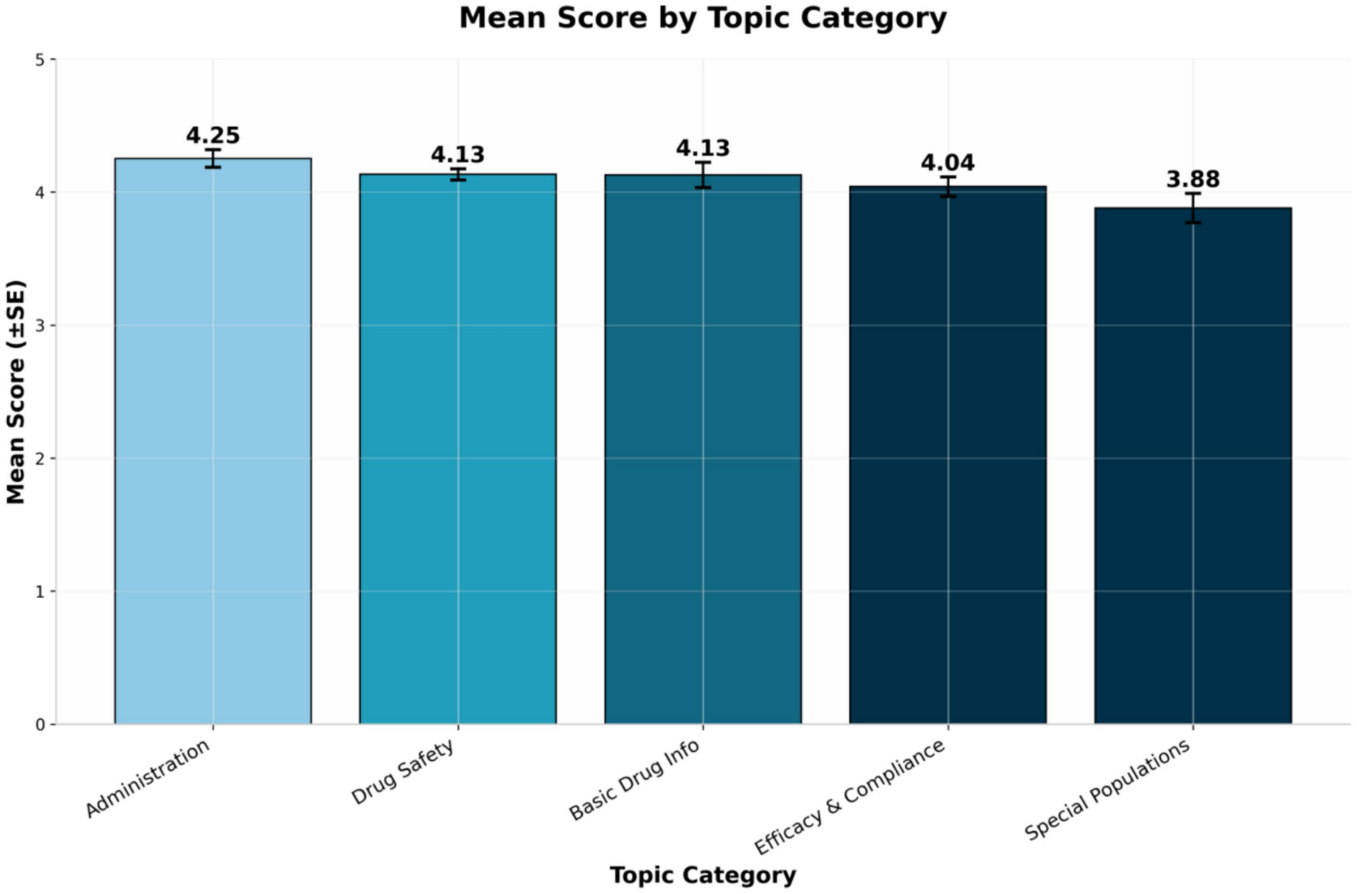
Bar chart of scores for different consultation topic categories. It clearly shows the score gradient of the five types of questions from highest to lowest, with the lowest score in medication use in special populations.

The scores of the five consultation topic categories ranked from highest to lowest were as follows: medication administration methods > drug safety > basic drug information > drug efficacy and medication adherence > medication use in special populations. The mastery level of medication administration methods was the best; medication use in special populations had high professional complexity and the lowest score. Figure 3 intuitively shows the score gradient differences among different topic categories.

### Inter-model correlation analysis

**Figure 4:**
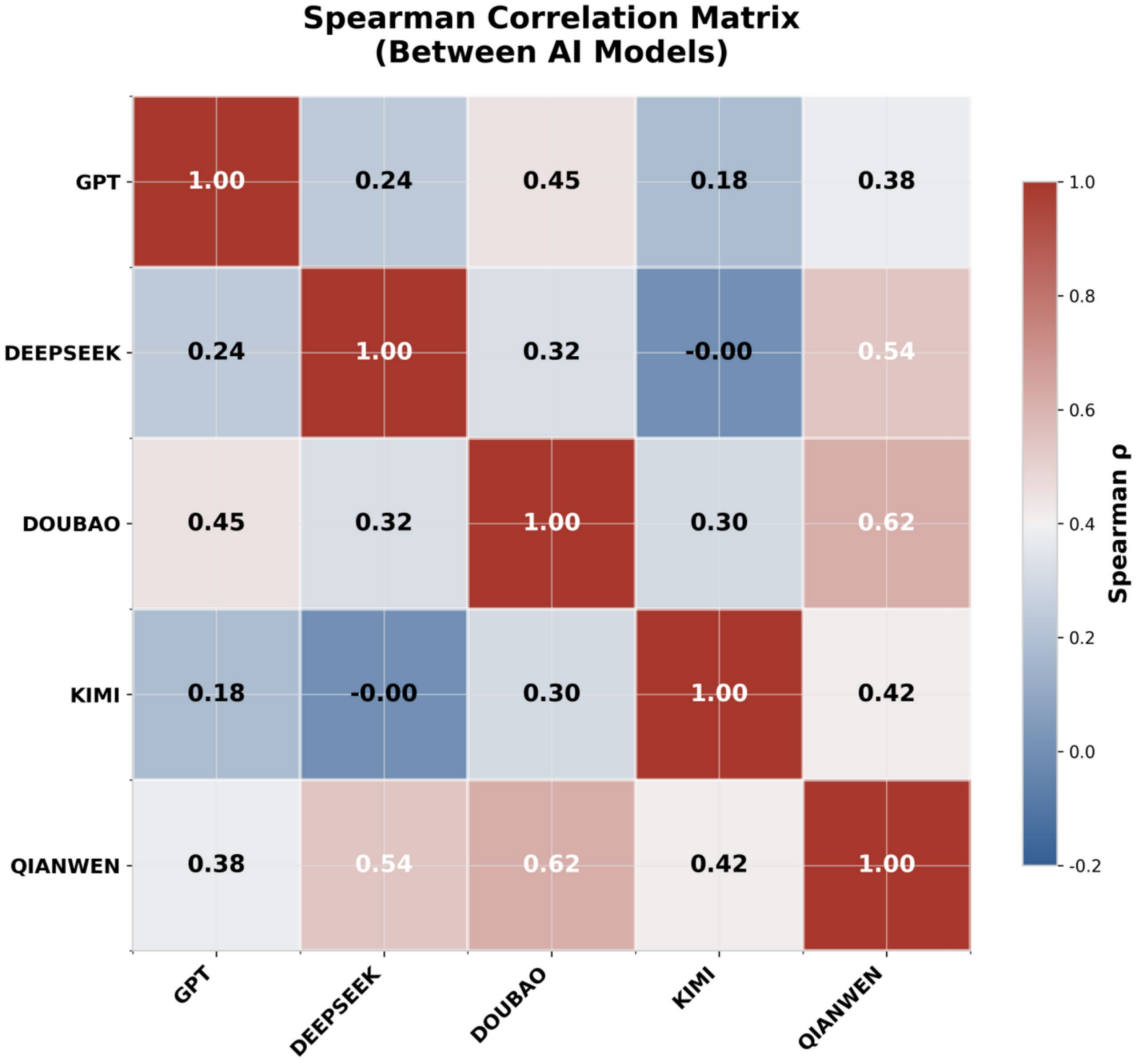
Spearman correlation heatmap among models. Colors and values represent the degree of correlation; the darker the red, the stronger the positive correlation, and the darker the blue, the stronger the negative correlation.

Spearman’s rank correlation analysis showed (Figure 4) that the correlation between Qwen and Doubao was the strongest (*r* = 0.62); Qwen had a moderate positive correlation with DeepSeek (*r* = 0.54); Kimi had almost no correlation with DeepSeek (*r* = 0.00), indicating complementary dimensional advantages; the other models showed weak to moderate positive correlations. The five-dimensional evaluation system had good discriminability.

### Comparative analysis of qualitative content differences in model answers

To provide concrete clinical context for the quantitative performance metrics, we selected illustrative examples from the model responses for qualitative analysis (Table 3). The examples were chosen to represent each model’s distinct strengths and weaknesses across different question types. This qualitative analysis, when combined with the quantitative data, offers a more comprehensive understanding of each model’s capabilities.

**Table 3:** Typical cases of qualitative comparison of model answers.

Questions	Core points of reference answers	ChatGPT	DeepSeek	Doubao	Kimi	Qwen
Dietary Requirements for Taking Ainuovirine	Take on an empty stomach; food increases plasma drug concentration and the risk of adverse reactions	Critical error: Can be taken with meals or on an empty stomach	Accurately indicated the requirement of taking on an empty stomach, and explained the mechanism and risks	Concise and clear indication of taking on an empty stomach, without redundancy	Accurately indicated taking on an empty stomach, without explaining the mechanism	Accurately indicated the requirement of taking on an empty stomach and explained the mechanism, with concise expression
Concomitant Use of Rilpivirine and Antacids	Do not take simultaneously; an interval of 4 h is required; antacids reduce drug absorption	Partial error: Recommended an interval of 2 h	Answered accurately and elaborated the mechanism and interval time completely	Clearly indicated that simultaneous administration is not allowed, and recommended an interval of more than 2 h	Accurately pointed out that simultaneous administration is not allowed and an interval of 4 h is required, with no deviation in core points	Answered accurately and explained the mechanism, with specific interval time
Management of Missed Dose of Biktarvy	Take the missed dose within 18 h; skip the missed dose if more than 18 h have passed; do not take a double dose	Vague expression: Only mentioned taking the missed dose as soon as possible, without time limit	Completely and accurately decomposed the management principles and clearly prohibited double dose	Clear logic, summarizing the core principles in one sentence	Accurately repeated the core principles, with no information deviation	Accurately provided the principles and attached practical operation tips

DeepSeek performed prominently in complex questions, demonstrating high accuracy and completeness, as exemplified by its detailed and accurate analysis of drug–drug interaction mechanisms. While its mechanism analysis was strong, some responses were noted to have slightly redundant expressions.

Qwen showed a high degree of question matching and strong reliability, providing concise and relevant answers without deviation. However, its completeness was slightly lower than DeepSeek’s.

Doubao exhibited clear logic and strong operability, with concise language, as seen in its step-by-step guidance for missed dose management. However, it occasionally lacked key information in a few complex questions, which is reflected in its moderate Completeness score in Table 2.

Kimi demonstrated the best stability, with no critical factual errors and a 100% matching degree of core points, as shown in its consistently accurate responses across various questions. This stability is corroborated by its high Accuracy score in Table 2, which was the highest among all models. However, its responses sometimes lacked detailed practical operation suggestions.

ChatGPT performed the worst overall, with frequent critical factual errors, insufficient basis, and imprecise reasoning, as dramatically illustrated by its incorrect statement about the dietary requirements for a specific antiretroviral drug. This qualitative observation is strongly supported by its significantly lower scores across all quantitative dimensions in Table 2, particularly its Accuracy and Reliability scores. It only performed relatively normally in simple factual questions.

These qualitative findings, supported by the examples in Table 3, directly corroborate and complement the quantitative performance differences observed in Table 2, providing a robust and multi-faceted evaluation of the LLMs.

## Discussion

### Analysis of overall performance of LLMs

Statistical analyses in this study reveal significant differences in the ability of the five LLMs to address HIV medication consultation queries. DeepSeek and Qwen demonstrate the best overall performance, Doubao stands out for its exceptional clarity, Kimi exhibits stable performance, while ChatGPT lags significantly behind the others. Chinese-developed models show distinct advantages in Chinese medical scenarios, adaptation to domestic clinical guidelines, and understanding of colloquial patient language (28). In contrast, international models exhibit clear limitations in specialized and localized pharmaceutical knowledge.

*DeepSeek*: its high accuracy and completeness stem from extensive training on specialized medical datasets (29), particularly those encompassing antiretroviral medication guidelines and drug package inserts. This enables precise dissection of complex drug–drug interaction mechanisms and comprehensive coverage of key query points. Its algorithm design prioritizes information integration and detailed presentation, resulting in significantly higher scores in the dimensions of completeness and clarity.

*Qwen*: the highest score in the clarity dimension reflects an algorithm design that emphasizes logical structure and conciseness, allowing for precise alignment with user intent and avoidance of topic deviation (30). The training data is well-annotated with references to clinical guidelines and drug package inserts, yielding a reliability score comparable to that of DeepSeek.

*Doubao*: responses are concise, well-structured, and aligned with the reading habits of clinical pharmacists and patients, offering strong clinical practicality. However, insufficient training on specialized data for certain complex queries results in a slightly lower completeness score.

*Kimi*: the core of its stable performance lies in rigorous quality control of training data, with no significant erroneous information incorporated, thus eliminating critical factual errors. Nevertheless, a lack of targeted algorithmic optimization for clinical practical recommendations leads to slightly inferior utility compared to other models.

*ChatGPT*: the frequent occurrence of critical errors is primarily attributed to severe insufficiency in specialized training data for HIV pharmacotherapy, particularly the absence of data on antiretroviral drugs marketed in China (e.g., Ainuovirine, Ainuomiti). This leads to incorrect responses regarding core information such as administration requirements and drug interactions. Additionally, its logical reasoning mechanism lacks rigor, with many statements lacking foundational evidence, resulting in an extremely low reliability score.

### Analysis of LLMs performance across different consultation types

The performance of LLMs across different types of consultation questions shows a consistent pattern: they perform well in factual questions but poorly in complex ones. Specifically, the models achieved the best performance in questions related to medication administration methods, while their performance was the worst in questions concerning special population medication use. This pattern is closely related to the characteristics of HIV medication consultation questions.

*Basic drug information*: this type of consultation is purely factual, involving inquiries such as indications, storage requirements, and active ingredients of drugs. The information is fixed and clear, and the models can directly match the required content from their knowledge bases without complex logical reasoning As a result, all models performed well in this category, with no critical errors, and could accurately provide the required information to meet the basic consultation needs.

*Drug safety*: this category mainly involves the management of adverse reactions and drug–drug interactions. Some content is factual and can be directly retrieved from the knowledge base, while other content requires in-depth analysis of the mechanism of action—for example, explaining why certain drugs cannot be used together and the potential risks involved. Models with strong comprehensive capabilities (such as DeepSeek and Qwen) could accurately decompose the mechanisms and provide clear explanations, while ChatGPT often gave incorrect answers due to insufficient professional data, failing to effectively address drug interaction issues.

*Special population medication and efficacy consultation*: these are practical questions that require combining individual patient conditions to provide personalized advice, such as pediatric dosage adjustments and missed dose management. Most models struggled to balance professionalism and practicality: some provided overly simplistic answers lacking actionable advice, while others included irrelevant content, failing to meet clinical practical needs (31–33).

*Medication administration methods*: represent the primary error-prone domain for ChatGPT. This is because such inquiries involve specialized content including pharmacokinetic characteristics related to drug absorption and metabolism, as well as the effects of food and dosing timing on therapeutic efficacy. Additionally, the scarcity of relevant research data for certain antiretroviral drugs marketed in China significantly increases the difficulty of model training, making this category a core challenge for ChatGPT in addressing HIV medication consultation (34, 35).

### Analysis of model errors and hallucinations

Despite the overall performance of the five LLMs, various types of model errors and hallucinations were observed during evaluation, which may threaten medication safety for people living with HIV. Three typical examples are summarized below.

First, factual errors in core administration instructions occurred in multiple models. For example, in response to the question “What are the dietary requirements for taking ainovirine?” ChatGPT incorrectly claimed that ainovirine “can be taken with meals or on an empty stomach,” which directly contradicts the official instruction that it must be taken on an empty stomach. Such errors may increase adverse reactions and treatment failure.

Second, hallucinated drug interaction advice was identified. When asked “Can rilpivirine be taken with antacids?” Doubao provided an inaccurate interval of 2 h, while the correct and safe interval required by clinical guidelines is 4 h. This fabricated unsafe advice poses a real risk of reduced drug efficacy.

Third, vague or evasive reasoning lacking clinical specificity was regarded as a mild form of hallucination. For the question “How to manage a missed dose of Biktarvy?” ChatGPT only repeated “take it as soon as possible” without mentioning the critical 18-h time window. This failure to provide evidence-based, actionable guidance represents incomplete and misleading output.

These cases demonstrate that LLMs still face challenges in specialized HIV pharmaceutical consultation, including inaccurate professional knowledge, fabricated safety intervals, and vague clinical instructions. Therefore, all models require strict expert review before clinical application.

### The impact of LLMs on pharmaceutical care

The findings of this study carry important implications for pharmaceutical care. Currently, LLMs demonstrate significant potential as auxiliary tools in the field of HIV medication consultation, yet their practical clinical application is accompanied by multiple challenges and opportunities.

*Enhancing service accessibility and efficiency*: as a chronic infectious disease requiring lifelong treatment, HIV/AIDS is characterized by high-frequency, long-term, and complex medication consultation needs among patients (36). Traditional pharmaceutical care models are constrained by human resources and temporal–spatial limitations, making it difficult to meet patients’ demands for round-the-clock, personalized medication guidance. Leveraging their advantages of instant response, multi-turn interaction, and knowledge integration, LLMs can effectively fill this service gap, providing patients with 24/7 uninterrupted medication consultation services and significantly reducing the risks of treatment non-adherence and failure caused by inappropriate medication use (37).

*Optimizing clinical decision support*: in prescription review and drug–drug interaction screening, high-performing models (e.g., DeepSeek, Qwen) have exhibited accuracy and completeness approaching that of clinical pharmacists. In the future, establishing a human-machine collaboration model of “LLM preliminary screening + pharmacist review” can free pharmacists from repetitive, basic consultation tasks, allowing them to focus more on formulating personalized medication regimens for complex cases and patient education, thereby elevating the overall quality of pharmaceutical care (38).

*Promoting equitable resource distribution*: medication education materials generated by LLMs feature standardization, structuring, and easy accessibility, serving as supplementary resources for HIV prevention and treatment in primary healthcare institutions and remote areas. This holds substantial practical significance given China’s current situation of over 1.3 million PLWH and uneven distribution of medical resources across regions (39).

However, it must be clearly recognized that LLMs cannot fully replace professional medical personnel. In this study, all models exhibited varying degrees of deficiencies in complex medication decision-making (e.g., dose adjustment for special populations, assessment of polypharmacy interactions), with some models even producing critical factual errors. Therefore, clinical deployment should strictly adhere to the principle of “LLM assistance, human decision-making,” and a robust answer review mechanism and error feedback channel should be established to ensure patient medication safety.

### The impact of LLMs on PLWH

PLWH are characterized by the uniqueness of their disease, social sensitivity, and the lifelong nature of their treatment. The application of LLMs in this field requires special attention to their value in providing psychological support, protecting privacy, and promoting treatment adherence.

*Reducing stigma and lowering consultation barriers*: PLWH often avoid in-person medical visits and consultations due to social stigma (40). The anonymous, non-judgmental interactive environment provided by LLMs can effectively lower the psychological barrier for patients seeking medication assistance, encouraging them to proactively consult about medication-related issues and thereby improving treatment adherence. This value is corroborated by the qualitative analysis in this study—the excellent performance of models in the “clarity” dimension (mean score: 4.53) indicates that patients can more easily understand medication guidance, reducing anxiety and self-doubt caused by ambiguous information.

*Strengthening medication education and adherence*: HIV treatment involves complex ART regimens, and patients must master accurate knowledge regarding dosing schedules, dietary requirements, and management of missed doses (41). This study demonstrates that models such as DeepSeek and Qwen exhibit outstanding performance in complex scenarios including missed-dose management and drug–drug interaction screening. They can generate personalized recommendations with specific time points and operational steps, which has direct clinical value in enhancing patients’ self-management capabilities and reducing the risk of drug resistance.

*Promoting doctor-patient collaboration*: LLMs can serve as a bridge connecting patients with professional medical teams. By recording patients’ consultation histories and identifying frequently asked questions, LLMs provide clinical pharmacists and physicians with data-driven insights into patients’ knowledge gaps regarding medication use, thereby optimizing subsequent in-person follow-up and health education strategies (42).

However, it is imperative to remain vigilant that the potential harm caused by model errors may be amplified for PLWH (43). Due to the high level of trust patients place in LLM-generated responses, errors in critical medication information (e.g., drug dosage, contraindications) could lead to treatment failure, the development of drug resistance, or even life-threatening consequences. Therefore, for this special population of PLWH, the application of LLMs must be accompanied by more rigorous accuracy verification mechanisms and explicit human review protocols.

### Limitations

*Limited scope of consultation questions*: this study only selected 55 common HIV medication consultation questions, and did not cover complex medication scenarios involving extreme cases, rare antiretroviral drugs, or patients with multiple comorbidities. The performance of LLMs in these scenarios still requires further investigation.

*Disregard for model version updates*: all models tested in this study were fixed versions. As models are continuously updated and training data are supplemented, their performance may change. Future research is needed to continuously track the performance of iterated models.

*Subjectivity in Scoring Criteria*: although the scoring process employed single-blind rating and adjudication by an arbitrator, certain subjectivity remains in the dimensions of “clarity” and “reliability.” Future studies can introduce objective metrics (e.g., key point matching rate, evidence annotation rate) to further optimize the scoring system.

*Single-response design*: each question was queried only once per model in this study. While the use of a standardized prompt and fixed model versions helped control for some variability, a single response may not perfectly represent a model’s average performance on a given query. Future research could employ multiple query iterations per question to quantify output consistency and provide a more robust measure of model reliability.

## Conclusion

Significant performance differences were observed among the five LLMs in addressing HIV medication consultation questions. DeepSeek and Qwen achieved the best overall performance, Doubao stood out for exceptional clarity, while ChatGPT demonstrated the weakest performance across all evaluated metrics.

All models performed best in the dimension of clarity, whereas relevance and completeness emerged as the primary limitations. Additionally, models exhibited superior performance on factual questions compared with complex application-oriented questions.

LLMs can serve as valuable auxiliary tools for HIV medication consultation, particularly for simple information queries. However, they remain insufficiently reliable in complex medication scenarios and cannot fully replace professional healthcare providers. Future efforts should focus on enhancing training with specialized HIV pharmacology data, optimizing clinical reasoning algorithms, and establishing rigorous human review and feedback mechanisms to ensure clinical safety and accuracy.

## Data Availability

The original contributions presented in the study are included in the article/Supplementary material, further inquiries can be directed to the corresponding author.
